# Clinical Outcomes and Genetic Mutations in Turkish Patients with Type 1 Gaucher Disease: Insights from a Single-Center Study

**DOI:** 10.3390/jpm15030109

**Published:** 2025-03-12

**Authors:** Ali R. Çalışkan, Jasmin Weninger, Huseyin Kaçmaz, Eda Nacar, Emine Şahin Kutlu, Hüseyin Onay, Süleyman Bayram, Ali Canbay, Mustafa K. Özcürümez

**Affiliations:** 1 Department of Gastroenterology, Faculty of Medicine, Adiyaman University, 02100 Adiyaman, Turkey; drkacmaz02@gmail.com; 2 Department of Medicine, Knappschaft Kliniken Universitätsklinikum Bochum, Ruhr University, 44892 Bochum, Germany; jasmin.weninger@knappschaft-kliniken.de (J.W.); ali.canbay@knappschaft-kliniken.de (A.C.); mustafa.ozcurumez@knappschaft-kliniken.de (M.K.Ö.); 3 Department of Internal Medicine, Faculty of Medicine, Adiyaman University, 02100 Adiyaman, Turkey; eda.evci@hotmail.com (E.N.); eminesahin02@gmail.com (E.Ş.K.); 4 Department of Genetics, Multigen Genetic Diseases Center, 35535 Izmir, Turkey; onayhuseyin@gmail.com; 5 Department of Public Health Nursing, Faculty of Health Sciences, Adıyaman University, 02040 Adıyaman, Turkey; sbayram@adiyaman.edu.tr

**Keywords:** gaucher disease, lysosomal storage disease, GBA1-mutation, enzyme replacement therapy, glucocerebrosidase

## Abstract

**Background:** Gaucher disease (GD) is a rare autosomal recessive lysosomal storage disorder caused by mutations in the GBA1 gene, leading to deficient β-glucocerebrosidase activity. This results in the accumulation of glucocerebroside in macrophages, primarily affecting the liver, spleen, bone marrow, and bones. Understanding the clinical outcomes and genetic mutation profiles in specific populations, such as Turkish patients, is essential for optimized disease management and personalized therapy and preventing morbidity and mortality. **Method:** This retrospective study analyzed data from 29 Turkish patients with previously diagnosed type 1 GD at a single center between September and December 2023. Genetic analyses were performed to identify GBA1 mutations using next-generation sequencing. Genetic mutations were the primary criterion for diagnosing GD. Clinical features, treatment responses, and outcomes were evaluated. Clinical parameters included hematological findings, organomegaly, and bone involvement. Data were analyzed to identify potential correlations between genetic mutations and clinical manifestations. **Results:** This study included 14 male and 15 female patients, with a mean diagnosis age of 22.1 years. A significant family history was observed in 93% of cases, and 52% had consanguineous parents. Epistaxis (72%) was the most common pre-diagnosis symptom. Most patients received enzyme replacement therapy with 60 units/kg. Treatment led to significant improvements, including increased hemoglobin (21.1%), higher platelet count (86.1%), and reduced organomegaly (liver (10.02%), spleen (25.22%)). Genetic analysis identified seven mutations, with c.1226A>G (p.N409S) being the most frequent. **Conclusions:** This study highlights the spectrum of clinical outcomes and genetic mutations in Turkish patients with GD, emphasizing the variability in disease severity based on genotype. GD should be considered for patients with unexplained nosebleeds, hepatosplenomegaly, bone pain, weakness, or siblings or other family members with similar symptoms. The genetic analysis revealed considerable heterogeneity among patients, which indicates the necessity of observing this in the development of personalized treatment strategies. Future studies with larger cohorts and long-term follow-up are needed to further elucidate genotype–phenotype correlations in this population.

## 1. Introduction

Gaucher disease (GD) is a rare autosomal recessive lysosomal storage disease caused by mutations in the glucocerebrosidase (GBA1) gene located on chromosome 1q21, leading to a deficiency in the enzyme β-glucocerebrosidase. This enzymatic defect leads to the accumulation of glucocerebroside in the lysosomes of reticuloendothelial system cells, known as Gaucher cells [1], which infiltrate various tissues and organs, including the liver, spleen, bone marrow, and bones [2]. The clinical manifestations of GD are diverse, encompassing symptoms such as fatigue, bone pain, recurrent nosebleeds, and growth retardation in children. The most common clinical signs include cytopenia, skeletal complications, and hepatosplenomegaly, which have a substantial impact on the quality of life and overall health of affected individuals.

First described by Philippe Gaucher in 1882, the disease has traditionally been classified into three distinct types based on clinical presentation and neurological involvement, although current evidence suggests that GD is better understood as a phenotypic continuum rather than as separated categories [3]. Historically, type 1 or non-neuropathic GD has been considered the most common form, affecting approximately 1 in 57,000 individuals; it can occur at any age and is associated with varying degrees of anemia, thrombocytopenia, hepatosplenomegaly, and bone involvement [4]. In contrast, type 2 (infantile or acute neuronopathic GD) and 3 (chronic neuronopathic GD) have been associated with central nervous system involvement, leading to more severe clinical outcomes and early death [5,6]. Bone complications, such as osteopenia, fractures, and avascular necrosis, are among the leading causes of morbidity in GD patients [7,8]. However, the clinical manifestations of GD are highly heterogeneous, with overlapping features and a broad spectrum of severity that may have phenotypes ranging from asymptomatic to debilitating. This variability is influenced by both the specific GBA mutations, with the extent of lipid accumulation in affected tissues, and environmental factors. Over 200 GBAS mutations have been identified with specific mutations associated with varying degrees of disease severity. The clinical course may be related to the causal mutation, with heterozygosity for the c.1226A>G (p.N409S [p.N370S]) mutation generally associated with the non-neuropathic form of the disease, whereas homozygosity for the c.1448T>C (p.L483P [p.L444P]) mutation is often linked to neurological involvement [3]. Moreover, although the c.1226A>G and c.84dup (p. Leu29fs [84GG]) mutations are among the most frequently detected, there is no clear-cut correlation between these specific genetic variations and the overall disease severity [9].

The prevalence and mutation spectrum of GD vary significantly across different populations, necessitating population-specific studies to better understand the genotype–phenotype relationships and optimize patient care. Despite advances in enzyme replacement therapy (ERT) and substrate reduction therapy (SRT), early diagnosis of GD and personalized treatment strategies remain critical for improving outcomes [10]. Genetic testing and measurement of low glucocerebrosidase activity in leukocytes or fibroblasts remain the gold standard for diagnosis [11]. ERT and SRT have marked a paradigm shift in the management of GD, offering patients the potential to achieve substantial symptom relief and enhanced quality of life [12]. Nevertheless, despite these therapeutic advancements, late diagnoses and untreated cases still pose challenges, leading to irreversible complications and, in severe cases, early mortality.

In Turkey, the prevalence of GD and its genetic mutation spectrum remain relatively understudied compared to other populations. Understanding the clinical presentation and genetic characteristics of GD in Turkish patients is crucial for tailoring management strategies and improving outcomes. This study focuses on evaluating the clinical outcomes and genetic mutations profiles in Turkish patients diagnosed with GD at a single center. The analysis of pre- and post-treatment parameters, including hematological indices, biochemistry profiles, organ dimensions and bone density, we aim to provide a comprehensive overview of the disease’s impact and the efficacy of available therapies in this specific population.

Furthermore, the present study aims to compare Turkish GD patients with global GD cohorts to highlight any unique clinical or genetic differences that may exist. Previous studies have demonstrated significant variability in the prevalence of GD-associated mutations across different populations. For instance, the N370S mutation is highly prevalent among Ashkenazi Jewish populations, while the L444P mutation is more frequently observed in East Asian and North African cohorts [13,14]. However, the mutation spectrum in Turkish patients remains to be elucidated, as current data are limited. This study explicitly hypothesizes that Turkish GD patients may demonstrate unique genetic variations and clinical manifestations that differ from global trends, potentially influencing treatment efficacy and disease prognosis. Previous research has suggested that genotype–phenotype correlations can vary based on ethnic background and environmental factors, which may impact disease progression and response to ERT or SRT [3]. By addressing these aspects, we aim to contribute valuable insights to the global understanding of GD while improving patient management in Turkey. The identification of population-specific differences could also have implications for genetic screening programs and early diagnosis strategies, ultimately leading to better clinical outcomes for affected individuals.

## 2. Methods

### 2.1. Study Population and Clinical Characteristics

This retrospective analysis was conducted to the Adıyaman University Education and Research Hospital a single tertiary care center in the Southeastern Anatolia Region of Turkey. The present study was performed between September and December 2023 and included 29 patients who had previously been diagnosed with and followed for type 1 GD. Inclusion criteria required a confirmed diagnosis of type 1 GD and the availability of complete clinical and laboratory data; patients with incomplete records were excluded from the study. A complete case analysis approach was used for any remaining missing values in the selected cases. The diagnosis of GD was made primarily based on the identification of genetic mutations in the GBA1 gene [15] using next-generation sequencing (NGS) and by measuring reduced glucocerebrosidase enzyme activity in peripheral blood leukocytes. The study population included all age groups and both sexes and varying age groups who had complete clinical and laboratory data were included in the study (Table 1).

Baseline data were comprehensively collected, including:

— Demographics: Age, sex, age at diagnosis, age at treatment initiation, treatment duration, and drug dosages were retrieved from the hospital’s information system.
— Family Background: Data on the number of siblings, family history, and consanguinity were collected through structured interviews conducted by trained clinical staff using standardized questionnaires. Clinical parameters such as the history of epistaxis were also documented.
— Biochemical Parameters: Routine laboratory tests were performed to measure blood markers including hemoglobin levels, white blood cell counts, and platelet counts. These biochemical parameters were recorded at both baseline and follow-up using the same laboratory protocols to assess changes in the clinical course and to ensure consistency.
— Instrumental Parameters:
  ○ Radiological Assessments: Liver and spleen dimensions, as well as the presence of hepatic steatosis, were evaluated via ultrasonography. These examinations were centralized, being performed by an experienced radiology team using consistent, standardized protocols. Hepatomegaly was defined as a craniocaudal liver length greater than 14 cm, and splenomegaly as a spleen length greater than 12 cm.
  ○ Bone Mineral Density: All patients underwent bone mineral densitometry using dual-energy X-ray absorptiometry (DEXA) at our centralized radiology unit. Bone health was assessed by recording Z-scores to evaluate bone involvement, a common complication in GD. The same DEXA scanner and calibration protocols were used for all measurements to ensure standardization.

All procedures were conducted according to the ethical standards of the committee responsible for human experimentation (institutional and national) and the 2008 revision of the 1975 Declaration of Helsinki. This study was approved by the Adıyaman University Non-invasive Clinical Research Ethics Committee (protocol number: 2023/3-1), date of approval 14 November 2023, and informed consent was obtained from all participants.

### 2.2. Genetic Analysis

**Figure 1:**
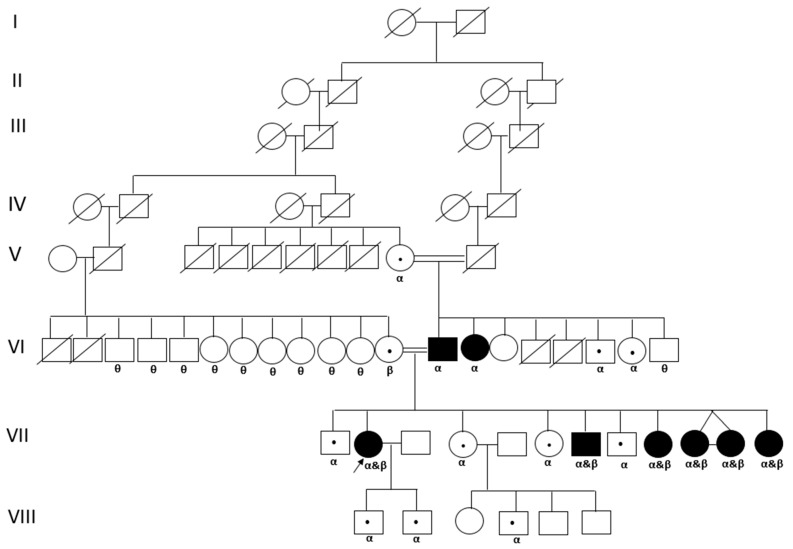
The pedigree of a family with GD type 1 with compound heterozygous c.1226A>G (p.N409S) and RecΔ55(c.1265_1319del) variant in the GBA gene. Circle: female. Square: male. An arrow marks the patient in our study. Affected GD individuals are represented by filled symbols and carrier of GD by a circle or square with a dot inside. Square and circle linked by a horizontal line represents marriage, a double horizontal line indicates consanguineous marriage. Deceased are illustrated by a circle or a square with a line running through it. Identical twins are displayed by a horizontal line between the siblings. **α:** Heterozygous c.1226A>G (p.N409S) [p.N370S] variant in the GBA gene; **β:** Heterozygous RecΔ55(c.1265_1319del) variant in the GBA gene; **Θ:** Genetic analysis could not be performed.

**Figure 2:**
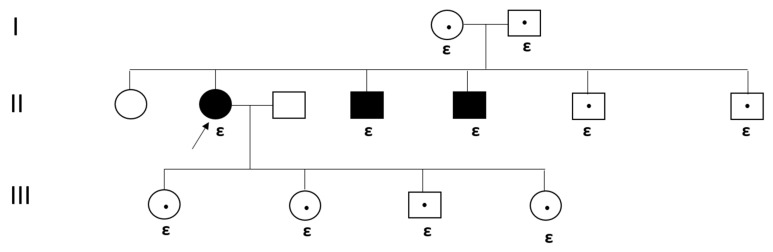
The pedigree of a family with GD type 1 with heterozygous c.1226A>G (p.N409S) variant and heterozygous c.1495G>A (p.V499M) variant in the GBA gene of a family with GD Type 1. Circle: female. Square: male. An arrow marks the patient in our study. Affected GD individuals are represented by filled symbols and the carrier of GD by a circle or square with a dot inside. Marriage: square and circle linked by a horizontal line. **ε**: Heterozygous c.1226A>G (p.N409S) [p.N370S]. These mutations were detected on the same allele (Cis mutation).

Genetic testing was performed on all patients to identify mutations in the GBA1 gene. Mutational analysis was conducted using next-generation sequencing, and variants were interpreted according to the American College of Medical Genetics and Genomics (ACMG) guidelines [16]. Patients were classified according to their genotype. Specific mutations such as p.N409S and RecΔ55 were investigated, with additional analyses performed for rare variants. In future analyses, the genotype–phenotype relationship will be further explored by correlating mutation types with disease severity and clinical outcomes using regression models that adjust for potential confounders (e.g., age, sex, and treatment duration). Figure 1 and Figure 2 illustrate two exemplary pedigrees of patients.

### 2.3. Treatment and Outcomes

Patients were only treated with ERT using Imiglucerase based on their individual clinical needs and treatment protocols. In the present study, the use of SRT or alternative treatments were not employed. Data regarding the type of treatment, its duration, and dosages were recorded. Pre-treatment and post-treatment parameters were compared, including hematological data, liver and spleen dimensions, and bone density scores (as measured by Z-scores) (Table 2). Response to treatment was classified as complete, partial, or non-responsive based on improvements in hematological indices, organ size, and symptom resolution.

### 2.4. Statistical Analysis

The collected patient data were analyzed via SPSS for Windows software (version 26.0; IBM Corp., Armonk, NY, USA). Descriptive statistics were used to summarize demographic and clinical data. Categorical variables are reported as frequencies (percentages), while continuous variables, such as hemoglobin levels, platelet counts, liver and spleen dimensions, and bone density scores, are reported as the means ± standard deviations (SDs), medians (interquartile ranges [IQRs]), and ranges. The normality of each variable’s distribution was assessed using the Shapiro–Wilk test. Since the variables followed a normal distribution, paired sample *t*-test were used to compare pre-treatments and post-treatment values. The relationships between continuous variables were evaluated using Pearson’s correlation coefficient (*r*). Statistical significance was set at *p* < 0.05. This comprehensive methodology aimed to provide insights into the clinical outcomes and genetic mutation profiles of Turkish patients with GD type 1, enabling a better understanding of the disease’s progression and treatment efficacy within this population.

## 3. Results

This single-center study evaluated 29 Turkish patients with GD, comprising 14 males (48%) and 15 females (52%). The mean age at diagnosis was 22.1 *±* 12.5 years, while treatment was initiated at an average age of 23.04 ± 11.79 years, and patients were enrolled in the study at 31.83 ± 12.24 years. A notable clinical symptom reported prior to diagnosis was nose bleeding, reported by 72% of patients, which is a lesser-known but potentially useful clinical sign in GD detection. A significant family history of GD was present in 93% of patients, and 52% had consanguineous parents, underscoring the importance of genetic predisposition and familial clustering in this population. The mean number of siblings per patient was 7.03 ± 2.61, of whom 2.83 ± 1.83 were also diagnosed with GD.

### 3.1. Treatment Outcomes

Of these, 23 patients (85.2%) were treated with a dosage of 60 units/kg, while 4 patients (14.8%) received 30 units/kg. Abdominal ultrasonography conducted during the study revealed hepatosteatosis in 11 patients (38%), with a mean grade of 1.09 ± 0.3 and a median grade of 1. This finding reflects an increased prevalence of liver involvement in GD patients, which may require monitoring over the course of treatment.

While the study revealed substantial improvements in several hematological and organ-related parameters following the initiation of Imiglucerase therapy, it is crucial to contextualize these findings in terms of clinical significance. Comparisons between measurements taken at diagnosis and those after treatment demonstrated statistically significant results for most parameters.

One of the most notable findings is the significant increase by approximately 21.1% in hemoglobin levels. At diagnosis, patients exhibited a mean hemoglobin concentration of 11.47 ± 2.23 g/dL, which increased to 13.89 ± 1.79 g/dL following treatment (t _(27)_ = −6.10, *p* < 0.001). Clinically, this suggests a meaningful reduction in anemia-related symptoms, such as fatigue and weakness, which substantially impact the quality of life of GD patients. Similarly, the platelet count showed a remarkable rise by about 86.1% from 91,689 ± 43,278 mcL at diagnosis to 170,620 ± 55,442 mcL after treatment (t _(28)_ = −8.85, *p* < 0.001), which is indicative of improved coagulation status and a lower risk of bleeding events. Organ enlargement, a hallmark of GD, was also significantly reduced. The liver size decreased from 17.07 ± 2.46 cm at diagnosis to 15.36 ± 2.02 cm after treatment (t _(27)_ = 4.27, *p* < 0.001), indicating a reduction of 10.02% in hepatomegaly. More prominently, spleen size exhibited a substantial decrease of approximately 25.22% from 18.72 ± 5.95 cm at diagnosis to 14.00 ± 3.28 cm after treatment (t _(26)_ = 5.62, *p* < 0.001). This improvement may correlate with a reduced risk of hypersplenism-related complications such as pancytopenia. Furthermore, growth and bone health improvements were reflected by the Z-score which improved from −2.12 ± 1.23 at diagnosis to −1.04 ± 1.22 after treatment (t _(18)_ = −3.24, *p* = 0.005), which represents an improvement of 50.94%, which is crucial given the significant burden of bone complications in GD.

### 3.2. Genetic Findings and Mutational Analysis

Genetic analysis of the GBA gene (NM_000157) in a cohort of 29 patients identified with seven distinct mutations. Among these individuals, 12 were found to be compound heterozygous for two different mutations, 10 were homozygous for a single mutation, and 7 exhibited double homozygous mutations. The identified mutations in the GBA are also presented in Table 3.

The specific mutations identified in this study were as follows:

1. Seven patients were homozygous for both the c.1495G>A (p.V499M) and c.1226A>G (p.N409S) variants.
2. Six patients were homozygous for the c.1226A>G (p.N409S) variant.
3. Six patients were compound heterozygous for the c.1226A>G (p.N409S) and RecΔ55 (c.1265_1319del) variants.
4. Four patients were homozygous for the c.1214G>C (p.S405T) variant.
5. Three patients were compound heterozygous for the c.1226A>G (p.N409S) and c.658T>A (p.S220T) variants.
6. Two patients were compound heterozygous for the c.1226A>G (p.N409S) and c.1193G>T (p.R398L) variants.
7. One patient was compound heterozygous for the c.1226A>G (p.N409S) and c.1214G>C (p.S405T) variants.

The most frequently observed mutation in the cohort was c.1226A>G (p.N409S), which appeared in both homozygous and heterozygous states. This mutation is well documented in GD and is associated with reduced enzymatic activity of glucocerebrosidase, contributing to disease pathogenesis. Additionally, the presence of other variants, such as RecΔ55 and c.1495G>A, underscores the genetic diversity among Turkish patients with GD.

## 4. Discussion

This study offers significant insights into the clinical characteristics, genetic spectrum and therapeutic outcomes of Turkish patients with GD, thereby contributing to the extant literature and shedding light on both the challenges and successes in managing this rare lysosomal storage disorder. The findings of the present study are in alignment with and serve to expand upon prior research, emphasizing the genetic underpinnings of GD, its phenotypic heterogeneity and the benefits of ERT with Imiglucerase.

### 4.1. Family History, Consanguinity, and Genetic Screening

The remarkably high prevalence of familial cases in this cohort (93% with a family history of GD) and the high rate of consanguinity (52% of parents were related) are consistent with reports from other populations with similar demographic patterns. For instance, a study by Sidransky [3] emphasizes the autosomal recessive inheritance pattern of GD, which is frequently amplified in populations with elevated rates of consanguineous marriages, notably in the Middle East, Mediterranean regions, and specific Jewish communities [17].

The mean of seven siblings per patient and an average of 2.83 affected siblings highlights the importance of early family-based genetic screening programs, a recommendation also made by Roshan Lal and Sidransky [18]. They argue that early identification of GD in at-risk individuals can prevent severe disease manifestations, as ERT has been shown to halt disease progression effectively when initiated early. However, in our study, the relatively late mean age at diagnosis (22.1 ± 12.5 years) underscores the need for increased awareness among healthcare providers to identify GD earlier in high-risk populations.

### 4.2. Clinical Presentation and Diagnostic Challenges

In this study, epistaxis (72%) was the most common presenting complaint, likely resulting from hematological manifestations of GD including thrombocytopenia. This finding is consistent with reports that bleeding tendencies and easy bruising [7] are hallmark early symptoms of GD [19]. Anemia as a debilitating symptom, contributing to fatigue, may be a component of pancytopenia resulting from bone marrow suppression or hypersplenism. Hepatosplenomegaly also leads to abdominal discomfort, significantly impacting patients’ daily activities and overall health. However, the absence of disease-specific symptoms frequently results in delayed or missed diagnoses, as was observed in this cohort. As Weinreb et al. have previously emphasized, delays in GD diagnosis are attributable to the disease’s nonspecific and variable presentation, particularly in regions with restricted access to specialized diagnostic facilities [20]. These delays are of particular concern due to the risk of irreversible complications, including bone disease, severe organomegaly, and growth impairment if the disease remains untreated. Public health initiatives, encompassing increased awareness campaigns and training programs for healthcare providers, are imperative to address these diagnostic challenges. This is particularly relevant in Turkey and other regions where consanguinity and large family sizes increase the genetic burden of GD.

### 4.3. Efficacy of Enzyme Replacement Therapy

The present study lends further support to the well-documented efficacy of ERT in improving the hematological, visceral, and growth parameters of GD patients, with significant improvements observed in hemoglobin levels, platelet counts, liver size, and spleen size. These observations are consistent with the findings of global studies, including those by Zimran et al. [21] and Grabowski [22]. These studies demonstrated that regular ERT with Imiglucerase leads to sustained improvements in anemia, thrombocytopenia, and hepatosplenomegaly, significantly improving patient outcomes and quality of life. The increase in Z-scores from −2.12 ± 1.23 to −1.04 ± 1.22 in our cohort highlights the positive effects of ERT on bone health, especially in pediatric and adolescent patients. This finding is consistent with the observations reported by Hruska et al., who noted that ERT not only reverses growth delays but also enhances bone density and reduces skeletal complications, which are of critical concern in GD [23]. Among these skeletal complications, the distal femur is the most affected bone, often exhibiting bilateral and symmetrical expansions in metaphysis and characteristic Erlenmeyer-shaped deformities on radiographs [24]. Over time, bone infarcts may develop, accompanied by osteopenia, osteonecrosis, and osteosclerosis, resulting from increased intraosseous pressure and vascular compression. These processes can trigger acute painful bone crises, known as Gaucher crises, which occur in 20–40% of patients and are more common in children. ERT has been shown to improve bone mineral density and reduce skeletal complications; however, pathological fractures, particularly in the femur, may still occur after the third decade of life [25].

It is interesting to note that, despite the clear benefits of ERT, hepatosteatosis was identified in 38% of patients. While the hepatosteatosis was generally mild (mean grade: 1.09 ± 0.3), its presence suggests that liver function should be closely monitored. This finding aligns with previous research indicating that some patients with GD may develop hepatic complications that persist despite ERT. Mistry et al. suggested that the accumulation of glucosylceramide and glucosyl sphingosine in hepatocytes may contribute to steatosis and other metabolic complications [26]. These findings emphasize the necessity for comprehensive liver monitoring during treatment and indicate that adjunct therapies, such as substrate reduction therapy, may be beneficial for patients with persistent hepatic involvement.

### 4.4. Genetic Spectrum of GD

This study identified seven distinct mutations in the GBA gene, reflecting the genetic heterogeneity of GD in the Turkish population. The most common mutation, c.1226A>G (p.N409S), was present in 25 of the 29 patients, either in homozygous or heterozygous form. This mutation, widely referred to as N370S in the literature, is known to be the most prevalent GBA mutation in non-neuronopathic GD and has been associated with a relatively milder phenotype [27].

Other mutations, such as RecΔ55 (c.1265_1319del) and c.1495G>A (p.V499M), highlight the genetic diversity within the cohort. Compound heterozygosity (41.4%) and double homozygosity (24.1%) were common, reflecting the complexity of the genotype–phenotype relationship in GD. This observation is consistent with findings by Beutler and Grabowski who reported that the severity of GD symptoms often correlates with specific GBA mutations, though environmental and other genetic factors also play a role [14].

A comparison of Turkish patients with Ashkenazi Jewish populations reveals a higher degree of genetic heterogeneity among the former, attributable to the predominance of the N370S (equivalent to c.1226A>G) mutation. The presence of mutations such as p.S405T and p.V499M, which are relatively rare in Western populations, may have distinct implications for disease severity and treatment response [3,14]. Further exploration in the form of functional studies and genotype–phenotype correlation analyses is warranted. In addition, given the growing recognition of genotype-driven therapeutic approaches, future research should assess whether certain mutations predict better outcomes with ERT versus SRT.

The identification of these mutations emphasizes the necessity for population-specific genetic studies to enhance comprehension of the mutation spectrum of GD across diverse geographical regions. For instance, the high prevalence of N370S in this cohort indicates its potential as a valuable marker for expedited genetic screening in Turkey, providing insights into genotype–phenotype correlations and facilitating earlier diagnosis and intervention. So, the interpretation of specific mutations has the potential to provide a valuable foundation for the development of prognostic tools and, indeed, the design of future personalized therapeutic interventions.

### 4.5. Implications for Clinical Practice and Research

The results of this study carry several implications for both clinical practice and future research. Firstly, they highlight the critical role of genetic counseling in the management of GD, particularly in populations with high rates of consanguinity. Counseling not only helps prevent disease transmission but also supports early identification of affected individuals, allowing for timely treatment. Given the high prevalence of consanguinity and familial cases, genetic counseling should be integrated into routine care for GD patients and their families.

Secondly, while ERT has been shown to be highly effective in reversing many of the systemic manifestations of GD and reinforcing the need for consistent and timely administration, the persistence of hepatic steatosis in a significant proportion of patients suggests that additional therapeutic approaches may be needed [28]. Emerging therapies, such as substrate reduction therapy and gene therapy, hold promises for addressing these unmet needs.

Finally, the study emphasizes the necessity for long-term follow-up studies to evaluate the impact of treatment on quality of life and long-term outcomes, including skeletal health, fertility, and the risk of malignancies, which are recognized complications of GD [26,29].

### 4.6. Limitations and Future Directions

Despite these strengths, the study has certain limitations, which merit consideration. First, the retrospective design of this analysis limits our ability to control potential confounders and may affect the robustness of the observed associations. Second, the relatively modest sample size (n = 29) restricts the extent to which the findings can be extrapolated to the broader Turkish population. This limitation is further compounded by the fact that all patients included in the study were treated exclusively with Imiglucerase. While this reflects the standard clinical practice at our center for managing GD, this selection criterion further reduces the already small sample size and consequently may not be directly applicable to patients managed with other alternative treatment options, such as substrate reduction therapies. Future studies should aim to include a broader range of therapeutic approaches in order to provide a more comprehensive evaluation of treatment outcomes across different patient populations with GD. Furthermore, the study did not assess the long-term impact of treatment on quality of life, an important consideration in chronic disease like GD. The lack of extended follow-up data limits our understanding of the sustained efficacy of therapy on daily functioning and well-being and potential late-onset complications. In light of these limitations, we recommend that future research should involve larger, multicenter, and ideally prospective or longitudinal studies to validate these results and provide a more comprehensive understanding of the clinical, genetic, and therapeutic landscape of GD in Turkey. Additionally, we suggest that practical recommendations be developed for genetic screening policies in Turkey, given the high prevalence of familial cases and consanguinity observed in our cohort. Such measures could facilitate earlier diagnosis and enable more personalized treatment approaches. Key recommendations include targeted screening for high-risk individuals (family history of GD, unexplained hepatosplenomegaly, anemia, or thrombocytopenia), voluntary carrier screening for couples, and potential inclusion of GD in newborn screening programs, using dried blood spot enzyme assays followed by confirmatory genetic testing. Expanding genetic testing infrastructure, strengthening the availability and affordability of next-generation sequencing, increasing awareness among healthcare professionals, and establishing a national GD registry are essential steps. Integrating GD screening into national health policies and collaborating with international organizations will further enhance disease detection and patient outcomes. Finally, future studies should also focus on exploring the genotype–phenotype relationship by correlating specific mutation types with disease severity and clinical outcomes. This approach may lead to the development of individualized therapeutic strategies that optimize patient care beyond the general efficacy of enzyme replacement therapy. These recommendations underscore the need for continued research in this area to ultimately improve the management and long-term outcomes of patients with GD.

## 5. Conclusions

In conclusion, this study provides important insights into the clinical, genetic, and therapeutic aspects of GD in Turkish patients. The findings reinforce the efficacy of Imiglucerase therapy in improving hematological and visceral parameters, such as anemia, thrombocytopenia, hepatosplenomegaly, and bone involvement, while highlighting the genetic diversity of GD in this population. These findings highlight the importance of early diagnosis and consistent long-term treatment in improving the quality of life for GD patients. However, given the limited sample size from a single center and the exclusive use of Imiglucerase, these results require validation in larger and more diverse cohorts with alternative therapeutic agents before broad clinical recommendations can be made. Moreover, the genetic analysis revealed considerable heterogeneity among patients, underscoring the complex genotype–phenotype correlations inherent to GD. While our results support the efficacy of ERT, they also highlight the potential for personalized treatment approaches based on individual genotype profiles rather than only a generalized application of ERT. Early diagnosis, targeted screening programs, and comprehensive genetic counseling remain critical for optimizing patient outcomes, particularly in regions with a high prevalence of familial cases and consanguinity. Future research should aim to validate these findings in lager cohorts and to focus on optimizing more tailored treatment strategies, understanding genotype–phenotype correlations, and addressing the persistent challenges of hepatic involvement and other long-term complications.

## Figures and Tables

**Table 1:** Patient characteristics.

	Category	*n*	%	Mean (SD)	Median (IQR)
**Gender**	Female	15	52%		
	Male	14	48%		
**Age at study enrollment (years)**		29		31.83 (12.24)	30 (24–35)
**Age at diagnosis** **(years)**		29		22.1 (12.5)	21 (14–25)
**Age at treatment initiation (years)**		27		23.04 (11.79)	24 (12–29)
**Number of children in the family**		29		7.03 (2.61)	7 (5–10)
**Number of sick siblings in the family**		29		2.83 (1.83)	2 (1–3)
**Receiving treatment**	Yes	27	93%		
	No	2	7%		
**Current treatment** **(mg/14 day)**		27		3422 (779)	3600 (2400–4400)
**Family history**	Yes	27	93%		
	No	2	7%		
**Related parents**	Yes	15	52%		
	No	14	48%		
**Nose bleeding at diagnosis**	Yes	21	72%		
	No	8	28%		
**Hepatosteatosis**	No	18	62%		
	Yes	11	38%		
**Hepatosteatosis grade**		11		1.09 (0.3)	1 (1–1)

**Table 2:** Comparison of measurements at diagnosis and after treatment.

	*n*	Mean (SD)	Median (IQR)	*t*	*p*
**Hemoglobin at diagnosis (g/dL)**	28	11.47 (2.23)	11.15 (9.95–13.05)	−6.10	<0.001
**Hemoglobin current (g/dL)**	28	13.89 (1.79)	13.72 (13.1–15.3)		
**Liver length at diagnosis (cm)**	28	17.07 (2.46)	17.0 (15–19)	4.27	<0.001
**Liver length current (cm)**	28	15.36 (2.00)	15.5 (14–17)		
**Platelet count at diagnosis**	29	91,689 (43,278)	81,000 (61,000–126,000)	−8.85	<0.001
**Platelet count current**	29	170,620 (55,442)	172,000 (120,000–208,000)		
**Spleen length at diagnosis (cm)**	27	18.72 (5.95)	17 (15–24)	5.62	<0.001
**Spleen length current (cm)**	27	14.00 (3.28)	14 (11–17)		
**T-score at diagnosis**	11	−1.44 (1.27)	−1.5 (−2.4–−0.7)	−0.40	0.698
**T-score current**	11	−1.29 (1.22)	−1.4 (−2.0–−0.8)		
**Z-score at diagnosis**	19	−2.12 (1.23)	−2.1 (−3.1–−1.4)	−3.24	0.005
**Z-score current**	19	−1.04 (1.22)	−1.2 (−2.0–−0.1)		
**Bone mineral density**	28	1.01 (0.18)	0.96 (0.88–1.16)		

**Table 3:** The identified mutations in the *GBA* gene.

Patient ♀/♂	Age	Homozygous for c.1495G>A (p.V499M) and c.1226A>G (p.N409S)	Homozygous for c.1226A>G (p.N409S)	Compound Heterozygous for c.1226A>G (p.N409S) and c.1265_1319 del (RecΔ55)	Homozygous for c.1214G>C (p.S405T)	Compound Heterozygous for c.1226A>G (p.N409S) and c.658T>A (p.S220T)	Compound Heterozygous for c.1226A>G (p.N409S) and c.1193G>T (p.R398L)	Compound Heterozygous for c.1226A>G (p.N409S) and c.1214G>C (p.S405T)
1. ♀	31	+						
2. ♀ *	30	+						
3. ♂ *	27	+						
4. ♂ *	28	+						
5. ♀ **	30	+						
6. ♂ **	32	+						
7. ♀ **	28	+						
8. ♂	27				+			
9. ♀	38				+			
10. ♀ ***	31				+			
11. ♂ ***	45				+			
12. ♀ ****	55		+					
13. ♂ ****	64		+					
14. ♂	44		+					
15. ♀	49		+					
16. ♂	25		+					
17. ♂	55		+					
18. ♀ *****	17			+				
19. ♀ *****	17			+				
20. ♀ *****	18			+				
21. ♂ *****	23			+				
22. ♀ *****	15			+				
23. ♀ *****	27			+				
24. ♂ ******	33					+		
25. ♀ ******	35					+		
26. ♀	32					+		
27. ♂ *******	24						+	
28. ♂ *******	22						+	
29. ♂	21							+
*n* (%)		7/29 (24.1%)	6/29 (20.7%)	6/29 (20.7%)	4/29 (13.8%)	3/29 (10.4%)	2/29 (6.9%)	1/29 (3.4%)

Siblings are indicated by different numbers of asterisks (*). Patient 17 is the father of the patients indicated by *****. Patients 18 and 19 are identical twins.

## Data Availability

The data supporting this study’s findings are available upon request from the corresponding author. The data are not publicly available due to privacy or ethical restrictions.
